# Obesogenic Dysregulation of Human Periprostatic Adipose Tissue Promotes the Viability of Prostate Cells and Reduces Their Sensitivity to Docetaxel and Cabazitaxel

**DOI:** 10.3390/medsci13040322

**Published:** 2025-12-16

**Authors:** Mariana Feijó, Lara R. S. Fonseca, Gonçalo Catarro, Cátia V. Vaz, Carlos Rabaça, Bruno J. Pereira, Eugenia Gallardo, Endre Kiss-Toth, Sara Correia, Sílvia Socorro

**Affiliations:** 1 RISE-Health, Department of Chemistry, Faculty of Sciences, University of Beira Interior, Rua Marquês d’Ávila e Bolama, 6201-001 Covilhã, Portugal; mariana@fcsaude.ubi.pt (M.F.);; 2 RISE-Health, Department of Medical Sciences, Faculty of Health Sciences, University of Beira Interior, Av. Infante D. Henrique, 6200-506 Covilhã, Portugalscorreia@fcsaude.ubi.pt (S.C.); 3 Laboratory of Drug-Toxicology, UBImedical, University of Beira Interior, Estrada Municipal 506, 6200-284 Covilhã, Portugal; 4 Instituto Português de Oncologia de Coimbra Francisco Gentil, Av. Bissaya Barreto, 3000-075 Coimbra, Portugal; 5 Faculty of Health Sciences, University of Beira Interior, Av. Infante D. Henrique, 6200-506 Covilhã, Portugal; 6 School of Medicine and Population Health, University of Sheffield, Sheffield S10 2RX, UK

**Keywords:** cabazitaxel, docetaxel, endocrine-disrupting chemicals, obesogens, periprostatic adipose tissue, prostate cancer

## Abstract

Background: Periprostatic adipose tissue (PPAT) has been shown to play a significant role in prostate cancer (PCa) development and progression. This relationship is further exacerbated by obesity, as PPAT-secreted factors increase PCa aggressiveness and have also been implicated in chemotherapy resistance. Therefore, identifying the molecular mediators of PPAT–prostate interorgan communication and the factors that disrupt this crosstalk is pivotal for better disease management. Obesogens, i.e., endocrine-disrupting chemicals that dysregulate adipose tissue towards an “obese” phenotype, have recently been implicated in disrupting this crosstalk, with an impact on prostate cell fate. Objectives: This study aimed to investigate whether obesogenic dysregulation of human PPAT secretory activity affects PCa cell viability and their response to docetaxel and cabazitaxel. Methods/Results: Through *ex vivo* culture of human PPAT and conditioned medium assays, we demonstrated that exposure to the model obesogen tributyltin (TBT) induced an “obese” phenotype in human PPAT, characterised by adipocyte enlargement and increased secretion of leptin and C-C motif chemokine ligand 7. The TBT-treated PPAT secretome enhanced cell viability and decreased the sensitivity of PCa cells to taxanes. Conclusions: This study provides preliminary evidence that lays the groundwork for future investigations, dissecting the molecular pathways underpinning prostate carcinogenesis and resistance to chemotherapy induced by obesogen-dysregulated PPAT.

## 1. Introduction

Periprostatic adipose tissue (PPAT) is the visceral fat depot that surrounds the prostate gland, directly contacting roughly one-third of the prostate’s anterior surface [1]. Recent research has demonstrated that in addition to its physiological functions related to energy storage and prostate mechanical protection [2], PPAT can significantly influence the behaviour of prostate cells. In the context of neoplasms, evidence indicates that PPAT–prostate inter-organ communication plays a significant role in driving prostate cancer (PCa) development and progression [1,3], which is further amplified in obesity, as “obese” PPAT presents heightened secretion of pro-inflammatory and tumorigenic factors [1,4], nurturing a pro-tumorigenic niche. Adipocyte-secreted factors, including adipokines (e.g., leptin) and chemokines (e.g., C-C motif chemokine ligand 7, CCL7), have been implicated as the main mediators of increased PCa aggressiveness [1,3,5,6]. An elegant study from Catherine Muller’s group demonstrated that obesity induces PPAT to secrete CCL7, which, in turn, promotes the directed migration of PCa cells expressing the C-C motif chemokine receptor 3 (CCR3), the CCL7 receptor, thereby facilitating extraprostatic extension and metastasis development [6].

The impactful role of PPAT in prostate carcinogenesis is underscored by evidence showing that PPAT is more effective than other adipose tissue depots in promoting PCa cell proliferation and migration [4]. This demonstration supports the concept of a highly specialised environment in which PPAT is biochemically and functionally adapted to sustain prostate tumorigenesis. Moreover, the recent clinical findings showing that PPAT thickness, as measured by magnetic resonance imaging, is associated with tumour upstaging after radical prostatectomy, with PPAT thickness >3 mm being predictive of more advanced disease [5], further corroborate the relevance of PPAT actions in mediating PCa development. Accordingly, a study showed that PPAT can induce chemotherapy resistance in PCa cells by upregulating the expression of antiapoptotic and cytoskeletal proteins through insulin-like growth factor-1 signalling [7]. This relationship is particularly relevant in the context of castration-resistant prostate cancer (CRPC), for which chemotherapeutic drugs such as taxanes are often the last line of treatment [8] yet frequently fail due to acquired resistance mechanisms influenced by the tumour microenvironment [9]. Therefore, identifying the key PPAT secretion products that trigger PCa onset, progression, and therapy resistance, as well as the factors that can deregulate PPAT function, will be pivotal for better disease management and improved patient clinical outcomes.

Obesogens are a subset of endocrine-disrupting chemicals (EDCs) characterised by their ability to reprogram adipose tissue towards an “obese” phenotype [10,11,12,13,14]. Over recent years, several governmental agencies and medical societies have expressed concern about the widespread harmful effects of EDCs, and specifically obesogens, on human health, as these chemicals can disrupt hormone signalling pathways, potentially contributing to the development of other diseases besides obesity, namely diabetes and hormone-dependent cancers [11,14,15,16,17,18]. Our research group recently demonstrated that the obesogen model tributyltin (TBT) dysregulated rat PPAT, with an impact on the fate of prostate cells, enhancing their survival by increasing viability, proliferation, and migration while reducing the apoptotic rate [19]. Considering the increasing environmental exposure to obesogens and the reported causal roles of these compounds in metabolic and cancer-related disorders [18,20], it is essential to better understand the depth of their effects in PPAT and beyond those observed in animal models. However, it remains unknown whether obesogens can affect human PPAT, leading to adipocyte dysfunction and disrupted crosstalk with prostate cells. In the present study, we aim to investigate whether obesogenic dysregulation of human PPAT secretory activity alters the viability of CRPC cells and affects their response to taxanes. We characterised the PPAT secretome in a set of PCa and benign prostate hyperplasia patients and the functional changes induced by the exposure to TBT. Thereafter, we evaluated how naïve PPAT and TBT-dysregulated PPAT (TBT-PPAT) influence prostate cells’ viability and their sensitivity to docetaxel and cabazitaxel.

## 2. Materials and Methods

### 2.1. Reagents

All reagents were acquired from Sigma-Aldrich (St. Louis, MO, USA) unless stated otherwise.

### 2.2. Human Samples

Human PPAT (*n* = 7) was obtained from patients submitted to radical prostatectomy or prostatic adenomectomy (Millin’s procedure) at the *Unidade Local de Saúde Cova da Beira* and *Instituto Português de Oncologia de Coimbra Francisco Gentil*, between September 2023 and January 2024, after approval of the research protocols by the hospitals and University of Beira Interior local ethics committees, and patients’ informed consent was obtained (CE-UBI-Pj-2022-033-ID1331, 9 May 2022). All procedures were performed in accordance with the Declaration of Helsinki. Each subject was attributed a number, and samples and all collected data were treated anonymously. Collected PPAT samples were maintained refrigerated in phenol red-free Dulbecco’s Modified Eagle Medium (DMEM, D1145), supplemented with 10% (*v*/*v*) foetal bovine serum (FBS, P30-193039, PANBiotech GmbH, Aidenbach, Germany) and 1% (*v*/*v*) penicillin/streptomycin/amphotericin B (A5955) until use in *ex vivo* culture procedures. Patients’ histopathological diagnosis, disease stage (if applicable), age, and anthropometric and other clinical data, such as the use of anti-cholesterol or -diabetic medication, are summarised in Table 1. 

Body mass index (BMI) was calculated, and patients were grouped as normal weight (BMI < 25 kg/m^2^), overweight (25 ≤ BMI < 30 kg/m^2^) or obese (BMI ≥ 30 kg/m^2^). Blood lipid (total cholesterol, low-density lipoprotein, LDL, and triglycerides), glucose, and free/total prostate-specific antigen (PSA) measurements are provided in Table 2.

### 2.3. Ex Vivo Culture of PPAT and TBT Treatment

PPAT (1–3 h after collection) was used for *ex vivo* culture and TBT treatment. Tissues were minced (2–3 mm^3^ fragments), washed in phosphate-buffered saline (PBS) for removal of impurities and blood vessels, and cultured (100 ± 5 mg tissue/mL) in DMEM without phenol red (D1145), supplemented with 10% (*v*/*v*) FBS (P30-193039, PANBiotech GmbH) and 1% (*v*/*v*) penicillin/streptomycin/amphotericin B (A5955) for 48 h. *Ex vivo* cultured PPAT was fixed in 4% paraformaldehyde (PFA, 100 ± 5 mg tissue/mL) for histological analysis. Cell culture media were collected and, upon centrifugation at 9700× *g* for 10 min, supernatants were stored in aliquots at −80 °C for posterior analysis and conditioned media (CM) assays. Alternatively, PPAT was treated with 100 nM TBT (T50202, CAS no: 1461-22-9) for 48 h in a humidified incubator at 37 °C with a 5% CO_2_-controlled atmosphere. TBT-treated PPAT (TBT-PPAT) and its culture media were used as described for control PPAT, i.e., histological analysis and CM assays. The chosen TBT concentration is in line with previous studies performed by us and others in adipocytes and prostate cells [23,24,25].

### 2.4. Histological Analysis

PFA-fixed PPAT and TBT-PPAT samples were embedded in paraffin, and sections (3 μm) were stained with haematoxylin and eosin (H&E), imaged at 400× magnification (Plan 40×/0.65 objective, Carl Zeiss, Gottingen, Germany) using an AxioImager A1 microscope (Carl Zeiss). To measure the size of adipocytes, the AxioVision Rel. 4.8 outline measurement tool (Carl Zeiss) was used. A total of thirty adipocytes per section was assessed across five random fields from each section.

### 2.5. Enzyme-Linked Immunosorbent Assays (ELISA)

Adipokines/chemokine levels in PPAT and TBT-PPAT culture media were measured using leptin (KAC2281, Invitrogen, Waltham, MA, USA), adiponectin (RAB0005), and C-C motif chemokine 7 (CCL7, RAB0078) sandwich ELISAs, following the instructions of the manufacturer. The sensitivity of each assay is 3.5 pg/mL, 25 pg/mL, and 20 pg/mL, respectively, for leptin, adiponectin, and CCL7. All incubation steps were performed at room temperature. For the high and zero standards, undiluted standard and diluent were used, respectively. Briefly, standards and samples were incubated for 2.5 h to allow interaction with the specific capture antibodies in pre-coated plates (except for leptin, where this step was performed simultaneously with the next), followed by a 1 h incubation with the biotin-labelled detection antibody (2 h for leptin). Then, HRP-streptavidin was added and incubated for 45 min (30 min for leptin). 3,3′,5,5′-Tetramethylbenzidine (TMB) chromogenic substrate was added in the dark and incubated for 30 min. After stopping the reaction (stop solution), the absorbance was immediately read at 450 nm using the xMark™ Spectrophotometer (Bio-Rad, Hercules, CA, USA).

### 2.6. Quantification of Extracellular Metabolites

Glucose (41010, Spinreact, Girona, Spain) and free fatty acids (FFAs, MAK044) levels in PPAT and TBT-PPAT culture media were determined spectrophotometrically using commercial kits according to the manufacturer’s instructions. For glucose quantification, the samples were mixed with the work reagent and incubated for 10 min at 37 °C. Absorbance values were measured at 505 nm (xMark™ Spectrophotometer, Bio-Rad). Regarding FFA quantification, according to the manufacturer’s protocol, samples were first incubated in the dark at 37 °C for 30 min with acetyl-CoA synthetase, followed by incubation with a reaction mix containing the fatty acid (FA) probe, enhancer, and enzyme mixture for 30 min at 37 °C. Absorbance values were measured at 570 nm (xMark™ Spectrophotometer, Bio-Rad).

### 2.7. Human Prostate Cell Lines

Non-neoplastic (PNT1A, [26,27,28,29,30]), neoplastic androgen-sensitive (22Rv1, [31]), and androgen-insensitive (DU145, brain metastasis, and PC3, bone metastasis of undifferentiated grade IV prostate adenocarcinoma, [32,33]) human prostate cell lines were purchased from the European Collection of Cell Cultures (ECACC, Salisbury, UK). Human prostate cell lines were cultured in DMEM with phenol red (D0822), supplemented with 1% (*v*/*v*) penicillin/streptomycin/amphotericin B (A5955) and 10% (*v*/*v*) FBS (P30-193039, PANBiotech GmbH), maintained in a humidified incubator (37 °C, 5% CO_2_-controlled atmosphere). The culture medium was replaced regularly (every 2–3 days), and subculture was performed whenever a confluence of 80–90% was reached. All experiments were performed using phenol red-free DMEM (D1145).

### 2.8. CM Assays and Chemotherapeutic Drug Treatments

Human prostate cell lines (PNT1A, 22Rv1, DU145, and PC3) were cultured for 24 h to allow cell adhesion and growth. After reaching a confluence of 60–70%, cells were cultured with different proportions of CM (10, 20, 30, and 60%) from untreated PPAT (PPAT-CM) or TBT-treated PPAT (TBT-PPAT-CM) for 24 h, and cell viability was assessed compared to the control group (standard culture media). DU145 and PC3 cells were cultured for another 24 h in the absence (control) or presence of the chemotherapeutic drugs docetaxel (7 nM) and cabazitaxel (1.5 nM), and viability was also evaluated.

### 2.9. Cell Viability Assays

Prostate cell viability was measured by the Sulforhodamine B (SRB, S1402) assay. The aminoxanthene dye SRB binds to protein basic amino acids and has been used as an indicator of proliferation. After the 24 h stimuli, cells were fixed in 10% cold trichloroacetic acid, followed by four washing steps with distilled water. After dryness at room temperature, SRB (0.05%) was added and the plate incubated in the dark for 1 h at room temperature. A solution of 1% acetic acid was used to remove unbound dye, while protein-bound dye was solubilised with Tris base solution (10 mM, pH 10). The absorbance was measured upon 10 min homogenisation at 540 nm (xMark™ Spectrophotometer, Bio-Rad), with the absorbance values being directly proportional to cell mass.

### 2.10. Gas Chromatography-Mass Spectrometry (GC-MS)

TBT levels in the TBT-PPAT-CM were determined by gas chromatography–mass spectrometry (GC-MS) in an HP7890B gas chromatograph coupled to a 5977A mass spectrometer (Agilent Technologies, Waldbronn, Germany). A 5% phenylmethylsiloxane-coated capillary column (30 m  ×  0.25 mm; 0.25 μm i.d., HP-5 MS, J & W Scientific, Folsom, CA, USA) was used to separate the analytes. Procedures were performed according to a modification described by Zachariadis and Rosenberg [34,35]. Briefly, 1.5 mL of sodium borohydride (4%, 452,882) and 6 mL of hexane were added to 500 µL of PPAT culture media (0 and 48 h of 100 nM TBT exposure) for further derivatisation and liquid–liquid extraction. For quantification purposes, tripropyltin (TPrT, 100 µg/mL, 8.08735) was used as an internal standard (IS). A 15 min room temperature derivatisation reaction was followed by rotation/inversion homogenisation for another 15 min. The organic layer containing the extracted tinhydrides was evaporated under a gentle stream of nitrogen until reaching approximately 100 µL. A 2 μL aliquot of the derivatised extracts was injected into the GC-MS system in splitless mode. The oven temperature, initially set to 30 °C for 1 min, was then increased to 280 °C at (30 °C/min), followed by a final hold at 280 °C (5 min). The mass spectrometer operated in the electron ionisation mode with a filament current of 70 µA. The inlet, ion source, and detector temperatures were maintained at 260 °C, 230 °C, and 280 °C, respectively. Helium served as the carrier gas (0.8 mL/min), and data acquisition was performed in selected ion monitoring (SIM) mode. The ions were chosen based on selectivity and abundance to maximise the signal-to-noise ratio in matrix extracts. The selected ions (retention times) were the following (quantifying ions italicised): 179, 235, and 121 *m*/*z* for TBT (7.15 min); 207 *m*/*z* for IS (6.92 min). DMEM media samples were prepared and analysed using the described extraction procedure in the range of 0.49–250 ng/mL (ten calibrators) for establishing the linearity of the method. Calibration curves were obtained by plotting the peak area ratio between TBT and IS. The acceptance criteria included a determination coefficient value (*R*^2^) ≥ 0.99 and a ±15% variation for calibrators’ accuracy. The lowest concentration that could be measured with adequate precision (≤20%) and accuracy ±20% variation, i.e., the lower limit of quantification (LLOQ), was 7.81 ng/mL (23.96 nM).

### 2.11. Statistical Analysis

A parametric unpaired *t*-test, one-way analysis of variance (ANOVA) followed by Tukey’s test, and two-way ANOVA followed by Sidak’s test were used to obtain the statistically significant differences between the tested groups, as applicable. The results were represented as mean ± standard error of the mean (SEM), considering the confidence interval of 95% and *p*-values < 0.05 as statistically significant. Statistical analyses were performed in the GraphPad Prism v8.0.1 software (San Diego, CA, USA).

## 3. Results

### 3.1. Characterisation of PPAT Morphology and Secretome

After collection, patients’ PPAT was analysed considering its morphological and secretory features, which is the foundational basis for an integrative interpretation of the results in the subsequent CM experiments.

**Figure 1:**
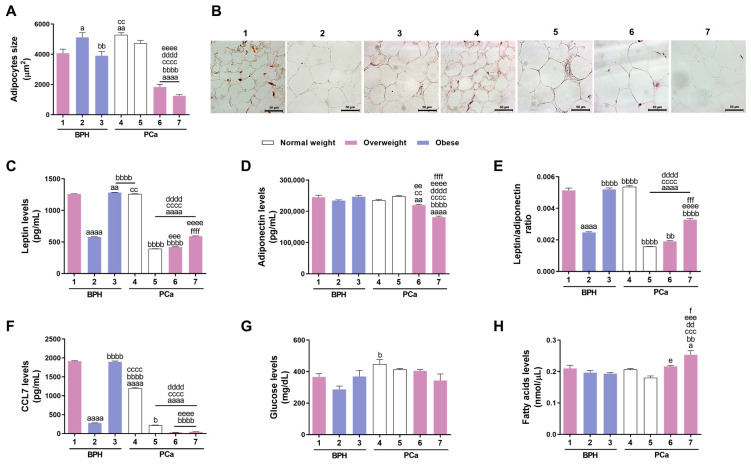
Morphology and secretome of naïve periprostatic adipose tissue (PPAT). PPAT from patients 1 to 7 was used for histological analysis and cultured *ex vivo* for 48 h for adipokines, cytokines, and metabolites quantification. (**A**) Adipocytes’ size measured using the AxioVision Rel. 4.6.3 (Carl Zeiss, Gottingen, Germany) after haematoxylin and eosin staining. A total of thirty adipocytes per section was analysed across a minimum of five random fields from each section. (**B**) PPAT representative images (Zeiss Axio Imager A1, Carl Zeiss). (**C**–**H**) Leptin (**C**), adiponectin (**D**), leptin/adiponectin ratio (**E**), C-C motif chemokine ligand 7 (CCL7, (**F**)), glucose (**G**), and fatty acids (**H**) concentration in PPAT culture medium. Data are presented as mean ± S.E.M. (triplicates of each sample, ANOVA followed by Tukey’s test). Statistically significant when compared to patient 1 (a; ^a^ *p* < 0.05, ^aa^ *p* < 0.01, ^aaaa^ *p* < 0.0001), patient 2 (b; ^b^ *p* < 0.05, ^bb^ *p* < 0.01, ^bbbb^ *p* < 0.0001), patient 3 (c; ^cc^ *p* < 0.01, ^ccc^ *p* < 0.001, ^cccc^ *p* < 0.0001), patient 4 (d; ^dd^ *p* < 0.01, ^dddd^ *p* < 0.0001), patient 5 (e; ^e^ *p* < 0.05, ^ee^ *p* < 0.01, ^eee^ *p* < 0.001, ^eeee^ *p* < 0.0001), and patient 6 (f; ^f^ *p* < 0.05, ^fff^ *p* < 0.001, ^ffff^ *p* < 0.0001). BPH: benign prostatic hyperplasia; PCa: prostate cancer.

Histological analysis showed that the size of adipocytes in the PPAT of patients 6 (P6) and 7 (P7) was significantly lower compared to other patients (P6: 1837.00 ± 173.40 and P7: 1250.00 ± 75.62 µm^2^ vs. 3902.00 to 5289.00 µm^2^ in the other patients; Figure 1A,B). The largest adipocytes were observed in P2, P4, and P5 (P2: 5124.00 ± 303.80, P4: 5289.00 ± 344.40 and P5: 4729.00 ± 466.30 µm^2^ vs. 1250.00 to 4075.00 µm^2^ in the other patients; Figure 1A,B).

ELISA and colorimetric assays were used to characterise the secretome of patients’ PPAT upon culture *ex vivo* for 48 h. Adipokines, such as leptin and adiponectin, chemokines, such as CCL7, and metabolites, such as glucose and FFAs, were the targets selected to be quantified as they are key players in prostate tumorigenesis and are differentially secreted by adipocytes in obesity [6,36,37,38,39,40,41,42,43]. Higher leptin secretion was found in P1, P3, and P4 PPAT (P1: 1259.00 ± 3.84, P3: 1283.00 ± 3.06, and P4: 1261.00 ± 2.60 pg/mL vs. 391.60 to 593.60 pg/mL in the other patients; Figure 1C). The lowest leptin secretion levels were detected in the PPAT of P5 and P6 (P5: 391.60 ± 1.53 and P6: 420.60 ± 3.79 pg/mL vs. 578.90 to 1283.00 pg/mL in the other patients; Figure 1C). Regarding adiponectin levels, the PPAT of P7 stood out the most, with significantly lower adiponectin secretion than that of other patients (181,063.00 ± 3641.00 vs. 219,963.00 to 248,330.00 pg/mL in the other patients; Figure 1D). Therefore, the results for the leptin/adiponectin ratio followed the differences observed in leptin secretion (P1: 5.15 × 10^−3^ ± 1.40 × 10^−4^, P3: 5.20 × 10^−3^ ± 9.00 × 10^−5^ and P4: 5.37 × 10^−3^ ± 6.00 × 10^−5^ vs. 1.58 × 10^−3^ to 3.28 × 10^−3^ in the other patients; Figure 1C,E). Marked differences were observed in the secretion of the CCL7 chemokine, which has been implicated in the crosstalk of PPAT with prostate cells, stimulating tumour development and invasion. PPAT from P6 and P7 presented a very low secretion of this chemokine (P6: 32.78 ± 1.11 and P7: 46.11 ± 1.11 pg/mL; Figure 1F), compared with the PPAT from P1, P3, and P4 which presented the highest CCL7 secretion (P1: 1915.00 ± 11.70, P3: 1896.00 ± 22.47, and P4: 1196.00 ± 17.46 pg/mL vs. 32.78 to 282.80 pg/mL in the other patients; Figure 1F). Glucose levels in PPAT secretome were similar among patients, with significant differences found only between P2 and P4 (respectively, 287.10 ± 21.12 vs. 447.40 ± 28.99 mg/dL; Figure 1G). The PPAT secretome from all patients presented similar FFAs’ content, which was only higher in P7 (0.25 ± 0.01 vs. 0.18 to 0.22 nmol/µL in the other patients; Figure 1H).

### 3.2. PPAT Secretome Slightly Increases Prostate Cell Viability

**Figure 2:**
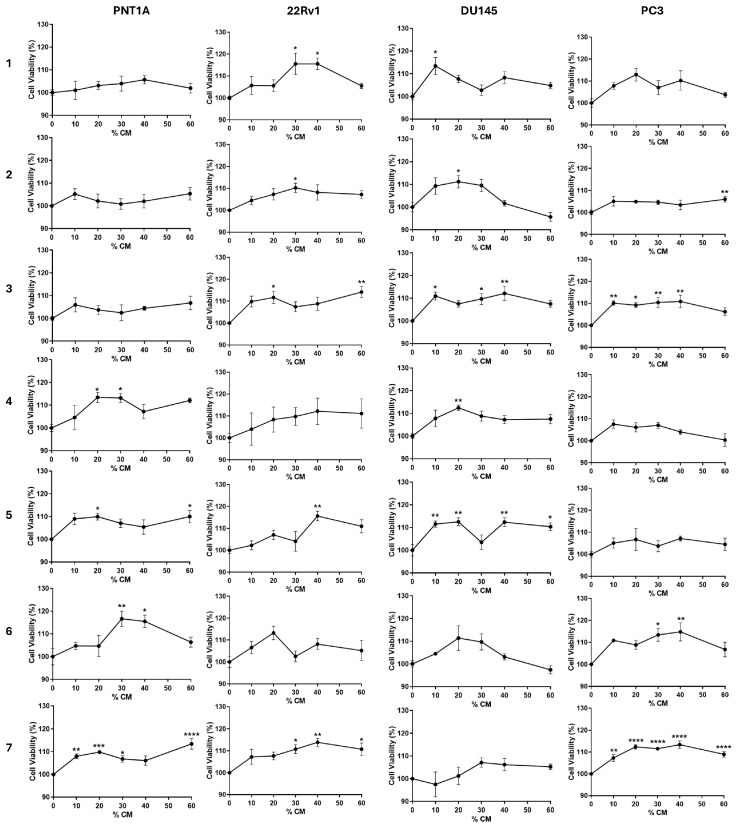
Effect of naïve periprostatic adipose tissue (PPAT) secretome (conditioned medium, CM) on the viability of human prostate cells. Non-neoplastic (PNT1A) and neoplastic androgen-sensitive (22Rv1) and androgen-insensitive (DU145 and PC3) cells were cultured with different percentages (0, 10, 20, 30, and 60%) of PPAT-CM from each patient (1–7) for 24 h, and cell viability was determined by the SRB assay. Results are expressed as a percentage relative to the DMEM control group. Error bars indicate mean ± S.E.M. (*n* = 6/group, ANOVA followed by Tukey’s test). Statistically significant differences relative to control are indicated as * *p* < 0.05, ** *p* < 0.01, *** *p* < 0.001, and **** *p* < 0.0001.

PPAT culture supernatants were collected after 48 h of *ex vivo* culture and used in CM assays to explore the ability of PPAT secretome to affect prostate cell viability. Non-neoplastic (PNT1A) and neoplastic androgen-sensitive (22Rv1) and androgen-insensitive (DU145 and PC3) cells were cultured with different proportions of PPAT-CM (0, 10, 20, 30, 40, and 60%) for 24 h, and cell viability was assessed by the SRB assay (Figure 2, Table 3).

The secretome of PPAT from P4, P5, P6, and P7 increased PNT1A cell viability, by 13.19–13.46, 9.94–10.00, 15.50–16.64, and ~6.09–13.43%, respectively, compared to control (Figure 2, Table 3). Regarding PCa cells, 22Rv1 cell viability was enhanced by the PPAT secretome of all patients, except P4 and P6 (P1: ~15.54–15.56, P2: ~10.23, P3: ~11.67–14.19, P5: ~15.62, and P7: ~10.75–13.90% higher viability than in the control; Figure 2, Table 3). DU145 cell viability was increased upon exposure to PPAT-CM from P1, P2, P3, P4, and P5 (respectively, ~13.45, ~11.22, ~9.71–12.16, ~12.47, and ~10.36–12.52% higher viability than in the control; Figure 2, Table 3), and in the case of PC3 cells, viability was increased in P2, P3, P6, and P7 (P2: ~6.00, P3: ~9.17–10.90, P6: ~13.43–14.83, and P7: ~7.31–13.41% higher viability than in the control; Figure 2, Table 3).

### 3.3. PPAT Secretome Reduced the Sensitivity of PCa Cells to Docetaxel and Cabazitaxel

Docetaxel and cabazitaxel were selected to investigate whether PPAT secretome contributes to chemotherapeutic drug resistance, considering that docetaxel-based chemotherapy is used in the treatment of CRPC and that, despite its initial effectiveness, most patients eventually develop resistance, requiring other cytotoxic approaches, such as cabazitaxel [44,45]. The CRPC cell lines DU145 and PC3 were cultured for 24 h in the absence or presence of patient PPAT-CM, followed by another 24 h in the absence (control, (-)) or presence of 7 nM docetaxel or 1.5 nM cabazitaxel. Selected concentrations align with previous studies, demonstrating the effectiveness of these two chemotherapeutic drugs in significantly reducing DU145 and PC3 cell viability [46,47,48].

**Figure 3:**
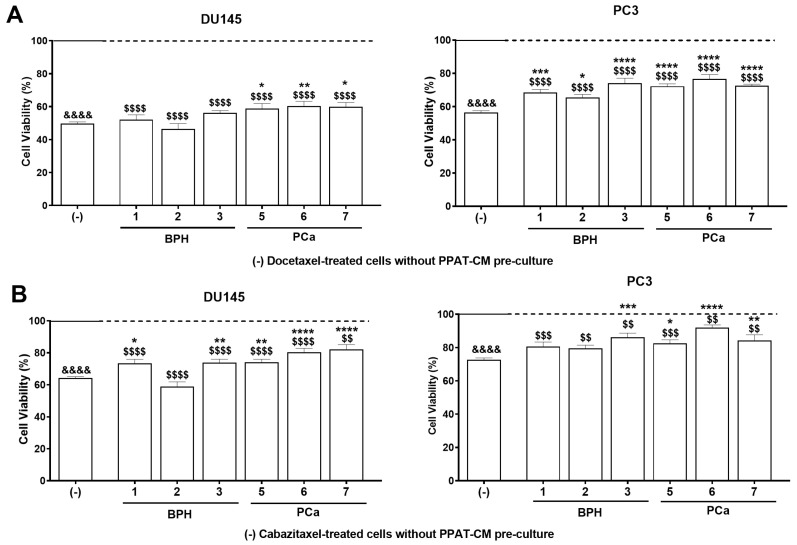
Effect of naïve periprostatic adipose tissue (PPAT) secretome (conditioned medium, CM) modulating the sensitivity of human PCa cells to chemotherapeutic drugs. DU145 and PC3 cells were cultured with 0% or 60% PPAT-CM from each patient (benign prostatic hyperplasia, BPH: 1–3 and prostate cancer, PCa: 5–7) for 24 h and then maintained for an additional 24 h in the absence or the presence of (**A**) docetaxel (7 nM) or (**B**) cabazitaxel (1.5 nM). Cell viability was determined by the SRB assay. (-) Cells treated with docetaxel or cabazitaxel without previous culture with PPAT-CM. (1–3, 5–7). Docetaxel- (**A**) and cabazitaxel-treated (**B**) cells after pre-culture with 60% PPAT-CM. Results are normalised and expressed as percentage relative to the respective control groups, i.e., DMEM for the (-) group (continuous line) and PPAT-CM for 1–3 and 5–7 (dashed line). Error bars indicate mean ± S.E.M. (*n* = 6/group, ANOVA followed by Tukey’s test). (*) Statistically significant when compared to the (-) group. (^&^) Statistically significant when compared to DMEM control group. (^$^) Statistically significant when compared to PPAT-CM control group. * *p* < 0.05, ** *p* < 0.01, *** *p* < 0.001, and **** *p* < 0.0001 and the same *p* values for & and $.

Docetaxel and cabazitaxel treatments were effective in significantly decreasing the viability of DU145 and PC3 cells (DU145: ~50.36 and ~35.78 and PC3: ~43.57% and ~27.4% less viability than in control, respectively; Figure 3). The PPAT-CM of all patients attenuated the docetaxel-induced reduction in PC3 cells’ viability in ~12.14, ~9.05, ~17.59, ~15.81, ~20.30, and ~16.13% for P1 to P3 and P5 to P7, respectively, while in DU145 this was only observed for P5, P6, and P7 (~9.08, ~10.80, and ~10.27% attenuation of docetaxel effect; Figure 3A). Regarding cabazitaxel, the PPAT-CM from all patients, except P2, was able to attenuate the cytotoxic effects of cabazitaxel for both cell lines (P1: ~9.14, P3: ~9.58, P5: ~9.96, P6: ~16.22, and P7: ~18.02% attenuation of cabazitaxel effect in DU145; P1: ~8.05, P3: ~13.61, P5: ~9.93, P6: ~19.30, and P7: ~11.61% attenuation of cabazitaxel effect in PC3; Figure 3B).

### 3.4. Exposure to TBT Alters PPAT Morphology and Secretome

Patients’ PPAT was cultured *ex vivo* in the presence or absence of 100 nM TBT for 48 h, a concentration and exposure time known to induce obesogenic effects [47]. Adipocytes’ size and PPAT secretome were evaluated to investigate the influence of TBT on these parameters.

**Figure 4:**
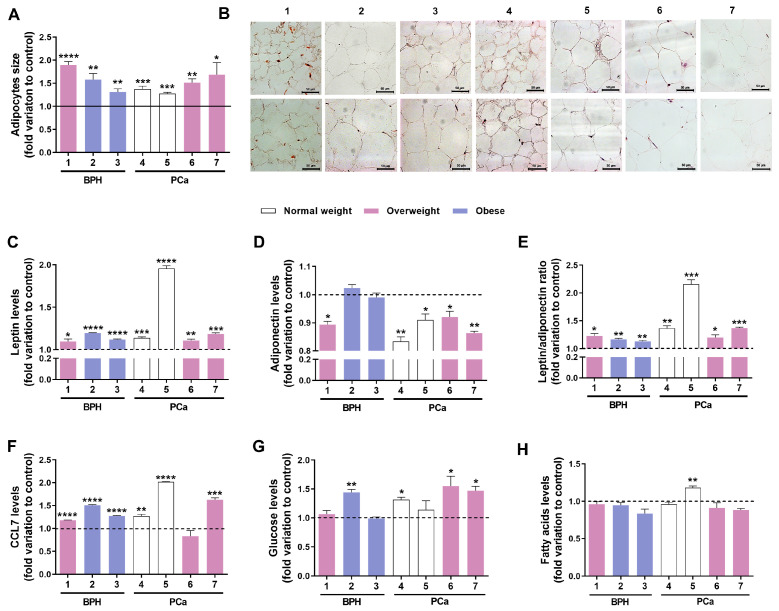
Tributyltin (TBT)-induced alterations in the morphology and secretome of human periprostatic adipose tissue (PPAT). Patients’ (1–7) PPAT was cultured *ex vivo* in the presence (100 nM) or absence (control) of TBT for 48 h. (**A**) Adipocytes’ size measured using the AxioVision Rel. 4.6.3 (Carl Zeiss, Gottingen, Germany) after haematoxylin and eosin staining. A total of thirty adipocytes per section were analysed across a minimum of five random fields from each section. (**B**) PPAT representative images (Zeiss Axio Imager A1, Carl Zeiss). (**C**–**H**) Leptin (**C**), adiponectin (**D**), leptin/adiponectin ratio (**E**), C-C motif chemokine ligand 7 (CCL7, **F**), glucose (**G**), and fatty acids (**H**) concentration in PPAT culture medium. Results are expressed as fold variation relative to the control (dashed line). Error bars indicate mean ± S.E.M. (triplicates of each sample, unpaired *t*-test). Statistically significant differences relative to control are indicated as * *p* < 0.05, ** *p* < 0.01, *** *p* < 0.001, and **** *p* < 0.0001. BPH: benign prostatic hyperplasia; PCa: prostate cancer.

Exposure to TBT promoted adipocyte hypertrophy in the PPAT of all patients, from 1.28- to 1.90-fold variation to control PPAT-CM (Figure 4A,B). Also, TBT significantly increased leptin secretion in all patients’ PPAT (1.10- to 1.96-fold variation to PPAT-CM; Figure 4C), with a highly accentuated effect in P5 (Figure 4C). TBT slightly reduced adiponectin levels in the PPAT secretome of all patients, except for P2 and P3 (0.92- to 0.83-fold variation to PPAT-CM; Figure 4D). Variations in the leptin/adiponectin ratio followed that of leptin (1.13- to 2.16-fold variation to PPAT-CM; Figure 4E). Upon TBT exposure, CCL7 levels were increased in the PPAT secretome of all patients, except P6, whose remained unchanged (1.18- to 2.02-fold variation to PPAT-CM; Figure 4F).

Regarding metabolic markers, TBT enhanced the glucose levels in the PPAT-CM of P2, P4, P6, and P7 (1.32- to 1.55-fold variation to PPAT-CM; Figure 4G). FFA secretion was altered only in P5 (1.18-fold variation to PPAT-CM; Figure 4H).

### 3.5. Exposure to TBT Enhanced the Effect of PPAT Secretome in Increasing Prostate Cell Viability

PPAT culture media was collected after 48 h of culture *ex vivo* in the presence or absence of 100 nM TBT (TBT-PPAT-CM) and used in 24 h CM assays to investigate whether TBT alters the ability of PPAT secretome to stimulate cell survival.

**Figure 5:**
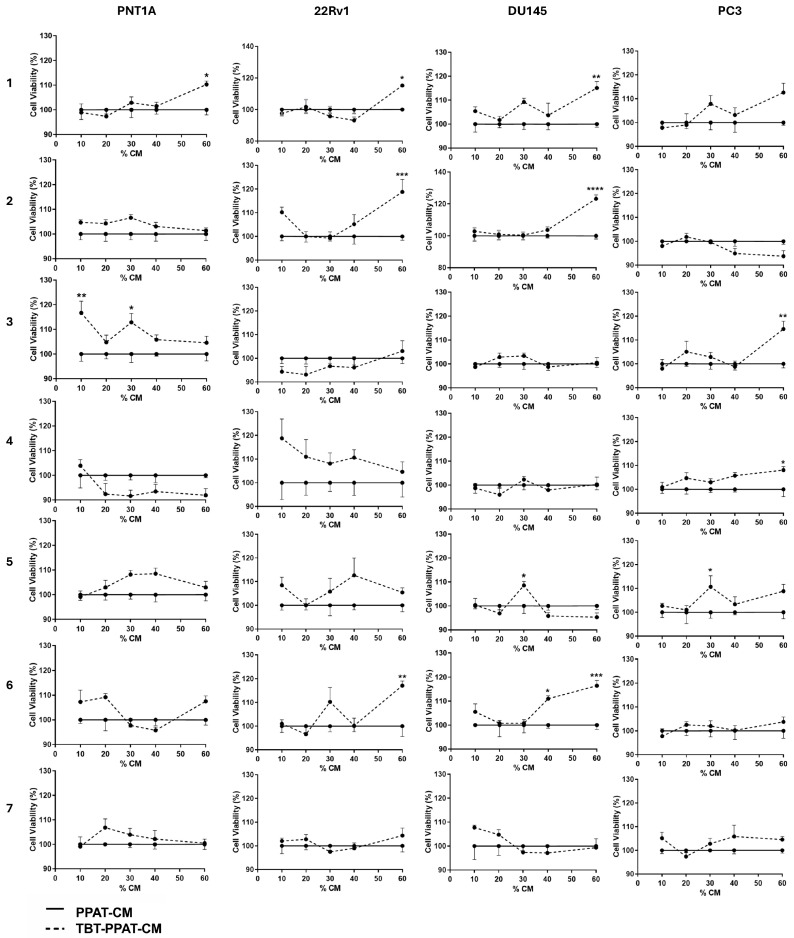
Tributyltin (TBT) effect in enhancing periprostatic adipose tissue (PPAT) secretome (conditioned medium, CM)-induced viability of human prostate cells. Non-neoplastic (PNT1A) and neoplastic androgen-sensitive (22Rv1) and androgen-insensitive (DU145 and PC3) cells were cultured with different percentages (0, 10, 20, 30, and 60%) of PPAT-CM or TBT-PPAT-CM from each patient (1–7) for 24 h, and cell viability was determined by the SRB assay. Results are expressed as percentage relative to the PPAT-CM control group. Error bars indicate mean ± S.E.M. (*n* = 6/group, ANOVA followed by Tukey’s test). Statistically significant differences relative to control are indicated as * *p* < 0.05, ** *p* < 0.01, *** *p* < 0.001, and **** *p* < 0.0001.

Overall, TBT enhanced the ability of PPAT secretome to increase prostate cell viability, except for P7 (Figure 5, Table 4).

TBT-PPAT-CM from P1 and P3 increased PNT1A cell viability, respectively, by ~10.28% and ~12.84–16.67% compared to PPAT-CM (Figure 5, Table 4). In the case of 22Rv1, cell viability was increased for TBT-treated P1, P2, and P6 PPAT (respectively, ~15.21, ~18.77, and ~17.11% higher viability than in PPAT-CM; Figure 5, Table 4). In androgen-insensitive PCa cells, TBT enhanced the effects of PPAT-CM from P1, P2, P5, and P6 in inducing DU145 cell viability (respectively, ~15.08, ~23.23, ~8.55, and ~11.01–16.44% higher viability than in PPAT-CM; Figure 5, Table 4). The same response occurred in PC3 cells with the TBT-PPAT-CM from P3, P4, and P5 (respectively, ~14.61, ~8.04, and ~10.68% higher viability than in PPAT-CM; Figure 5, Table 4).

**Figure 6:**
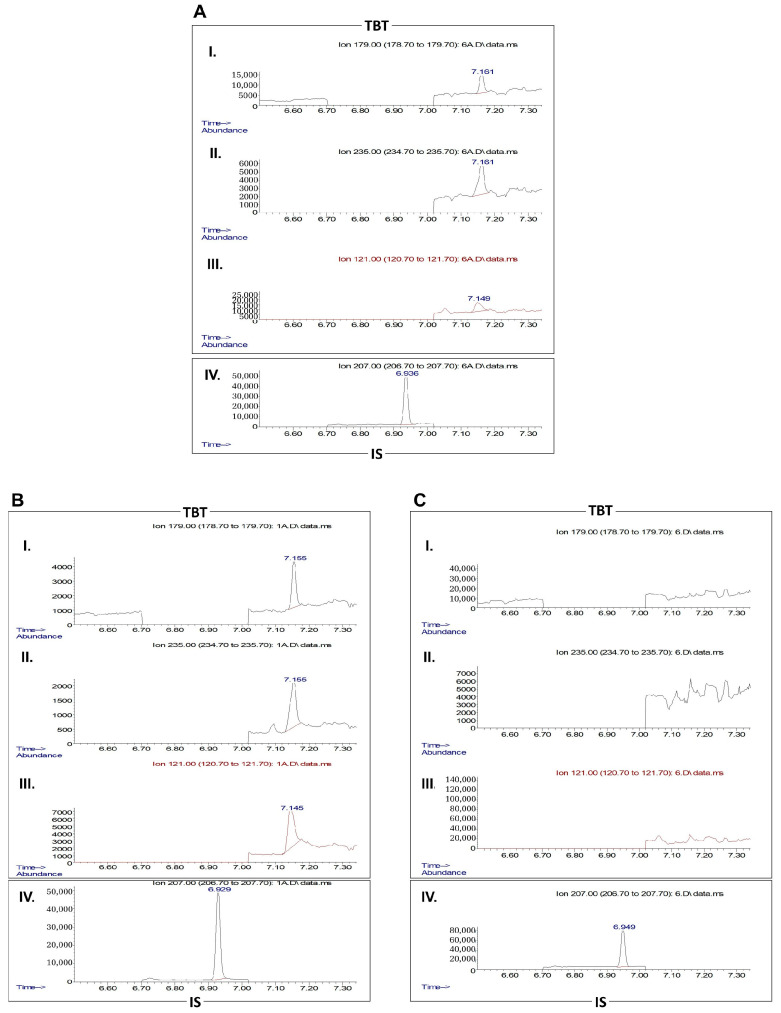
Chromatograms for tributyltin (TBT) and internal standard (IS, tripropyltin) in the periprostatic adipose tissue-conditioned media (PPAT-CM) in the course of the TBT experiments. PPAT was cultured *ex vivo* in the presence of 100 nM TBT for 48 h. (**A**) Lower limit of quantification (LLOQ) for TBT in DMEM medium (23.96 nM). (**B**) TBT at 0 h of the experiment (100 nM). (**C**) TBT after 48 h of culture (not detected). TBT quantifier (I. 179 *m*/*z*) and qualifier (II. 235 and III. 121 *m*/*z*) ions are represented, as well as the quantifier ion for the IS (100 µg/mL, IV. 207 *m*/*z*).

To confirm that the effects observed upon exposure to TBT-PPAT-CM were driven by the TBT-induced alterations in the PPAT secretome and not due to its residual presence, the compound levels in the CM used for prostate cell culture were quantified by GC-MS. TBT was not detected in the 48 h culture media of exposed PPAT (Figure 6).

### 3.6. TBT Effects on the Potential of PPAT Secretome in Reducing PCa Cells’ Sensitivity to Docetaxel and Cabazitaxel

**Figure 7:**
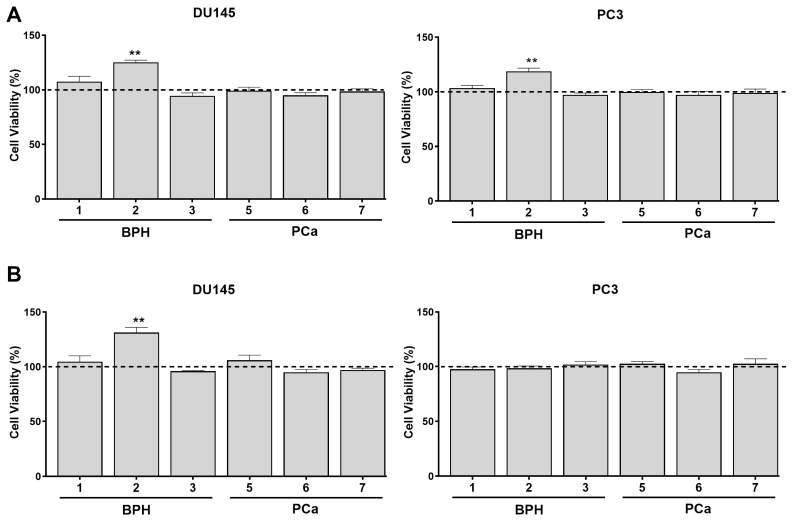
Tributyltin (TBT) effect in modulating the influence of periprostatic adipose tissue (PPAT) secretome (conditioned medium, CM) on the sensitivity of human PCa cells (DU145 and PC3) to chemotherapeutic drugs. DU145 and PC3 cells were cultured with 0% or 60% PPAT-CM or TBT-PPAT-CM from each patient (benign prostatic hyperplasia, BPH: 1–3 and prostate cancer, PCa: 5–7) for 24 h and then maintained for an additional 24 h in the absence or the presence of (**A**) docetaxel (7 nM) or (**B**) cabazitaxel (1.5 nM). Cell viability was determined by the SRB assay. Results are expressed as percentage relative to the respective PPAT-CM + drug group (dashed line). Error bars indicate mean ± S.E.M. (*n* = 6/group, unpaired *t*-test). Statistically significant differences relative to the respective PPAT-CM + drug group are indicated as ** *p* < 0.01.

To investigate the capability of TBT in modulating the effect of PPAT-CM attenuating the sensitivity to chemotherapeutic drugs, DU145 and PC3 cells were cultured for 24 h in the absence or presence of PPAT-CM or TBT-PPAT-CM, followed by another 24 h in the absence (control) or presence of 7 nM docetaxel or 1.5 nM cabazitaxel. Treatment with TBT only enhanced the ability of the P2 PPAT secretome in decreasing the sensitivity of CRPC cells to the studied chemotherapeutic drugs (Figure 7). Specifically, ~25.30% in DU145 and ~18.7% in PC3 for docetaxel, and 31.30% in DU145 for cabazitaxel (Figure 7).

## 4. Discussion

The present study aimed to investigate whether exposure to the environmental obesogen TBT alters the secretome and functionality of human PPAT and whether this has an impact on the viability of prostate cells and on their responsiveness to the chemotherapeutic drugs docetaxel and cabazitaxel. As a starting point, we characterised the morphology and secretome of the naïve PPAT from study participants and their effects on the viability and response of prostate cells to docetaxel and cabazitaxel. These analyses aimed to establish a foundational basis for an integrative interpretation of results following exposure of PPAT to TBT. Regarding adipocyte size, P6 and P7 presented smaller adipocytes than the other patients. This finding is consistent with previous reports showing that patients with type 2 diabetes mellitus, as is the case of P6 and P7, exhibit a higher percentage of small adipocytes [49]. Interestingly, in the subset of patients included in the present work, overweight was not a determining factor for the presence of larger adipocytes or altered adipokine secretion. A justification can lie in the tenuous discrepancy of the BMI values between study subjects. Patients with normal weight (P4 and P5) presented a BMI close to the limit to be considered as overweight (24.2 and 24.5 kg/m^2^, respectively, vs. the limit of 25 kg/m^2^), and the same tendence was verified in P2 and P3 obese patients (31.0 and 30.7 kg/m^2^, respectively, vs. the lower limit of 30 kg/m^2^). Moreover, similar levels of glucose and FFAs were detected in the PPAT secretome of the studied subjects, which indicates a comparable metabolic status. Notably, patients with higher leptin levels and a higher leptin/adiponectin ratio in PPAT secretome also showed higher CCL7 secretion (P1, P3, and P4). Although no direct evidence exists correlating CCL7 with leptin secretion by adipose tissue, CCL7 is a chemokine involved in recruiting monocytes to sites of inflammation, and its secretion is influenced by pro-inflammatory stimuli, namely TNF-α [50]. Given that leptin is strongly associated with inflammation, it is understandable that patients with higher leptin levels in the PPAT secretome (remitting for a pro-inflammatory state) are those that display higher CCL7 levels [51,52]. However, it is important to highlight that the small patient sample allows us to identify only hypothetical associations with patients’ characteristics, and further investigation in comprehensive patient cohorts is needed to establish correlations accurately.

Communication between PPAT and prostate cells, and PPAT’s influence on prostate cell fate, have been demonstrated, with PPAT-derived factors promoting the proliferation and invasiveness of PCa cells [4]. In general, and regardless of the different observed patterns, the viability of non-neoplastic (PNT1A: P4, P5, P6, and P7) and neoplastic androgen-sensitive (22Rv1: P1, P2, P3, P5, P7) and insensitive cells (DU145: P1, P2, P3, P5; PC3: P2, P3, P6, P7) was enhanced by the secretome of human PPAT. Notably, only the secretome of PPAT from PCa patients increased the viability of non-neoplastic prostate cells. This outcome led us to hypothesise that not only does PPAT stimulate prostate cell growth but a dynamic reciprocal interaction between PCa cells and PPAT can also occur. The PPAT shaped by growing PCa cells gradually adapts its behaviour and acquires a protumoral phenotype that could sustain cell viability and migration. Knowledge on this “reverse” relationship is scarce; however, the existing evidence, though limited, demonstrates that the secretome of PCa cells can modulate adipokine secretion by PPAT, indicating the pervasive extent to which tumour cells command PPAT to produce factors that would stimulate their aggressiveness [53].

Regarding responsiveness to chemotherapeutic drugs, PPAT-CM from all patients attenuated docetaxel-induced suppression of PC3 cells viability. In DU145 cells, only the PPAT-CM from patients with prostate carcinoma (P5-P7) had the same effect, worsening the response to docetaxel, further supporting the aforementioned idea that PCa cells can communicate bidirectionally with PPAT. Presently, there is no definitive explanation for the broad effects observed in PC3 cells vs. DU145 cells, only with cancer-associated PPAT-CM. However, as PC3 cells represent a more aggressive stage of PCa than DU145, they may be more prone to drug resistance and to the actions of PPAT’s secreted factors in attenuating docetaxel effects.

In the case of cabazitaxel, the PPAT-CM of all patients, except P2, attenuated its cytotoxic effects in both cell lines. Noteworthy, the PPAT secretome was more effective in reducing sensitivity to cabazitaxel than to docetaxel. These results are particularly worrisome, as cabazitaxel is used as a later-line treatment for docetaxel-resistant tumours. The mechanism of action of cabazitaxel resembles docetaxel in binding to tubulin and stabilising microtubules, but cabazitaxel has lower affinity for the P-glycoprotein efflux pump [54]. Thus, resistance to docetaxel develops due to the overexpression of βIII-tubulin and P-glycoprotein, whereas cabazitaxel remains effective in these conditions, making it a valuable option against docetaxel-resistant tumours [54]. Our observations lead us to hypothesise that PPAT secretome induces significant molecular alterations that could sustain the reduction in PCa cells’ sensitivity to these chemotherapeutic agents, with a greater magnitude in the case of cabazitaxel and likely independent of the expression of βIII-tubulin and P-glycoprotein. This hypothesis, generated by our *ex vivo* study, is foundational for establishing new research objectives to be explored in detail in future studies aimed at clarifying the contribution of PPAT to treatment resistance in PCa. Exposure to TBT promoted adipocyte hypertrophy and increased leptin secretion in the PPAT of all patients. In contrast, it reduced adiponectin levels in the PPAT secretome of all patients (non-significant in P2 and P3). In the case of energy metabolites, TBT increased glucose levels in the PPAT-CM of P2, P4, P6, and P7 and FFA levels in P5. The alterations induced by TBT in PPAT morphology and secretome are in line with those previously reported by us in PPAT [19] and others in other types of visceral adipose tissue [55]. Leptin, glucose, and FFAs are proven pro-carcinogenic molecules, with their enhanced presence in the PPAT secretome and, consequently, in the periprostatic environment posing a risk for PCa development and aggressiveness. Leptin, a hormone primarily produced by adipose tissue (adipokine), has elevated levels associated with increased PCa risk and progression by stimulating cell proliferation, migration, and invasion [56,57]. Concerning energy metabolites, increased glucose and FFAs can also stimulate PCa development. Cancer cells often exhibit enhanced glycolytic flux and preferentially rely on glycolysis for ATP production, and higher levels of FFAs were found in the serum of PCa patients, with *in vitro* experiments demonstrating that high FFA levels can stimulate the proliferation, migration, and invasion of PCa cells [58].

The presence of TBT also increased CCL7 levels in the PPAT secretome, an effect observed in all patient PPATs except P6, consistent with our previous demonstration of the capacity of this obesogen to deregulate chemokine signalling throughout the PPAT–prostate cell communication axis [19]. CCL7 has been reported to have pro-tumorigenic actions by facilitating the migration and invasion of prostate cells in an interplay with the PPAT [6,19]. This is mediated through the CCL7 receptor, C-C motif chemokine receptor 3 (CCR3), where the CCL7 secreted by the PPAT creates a paracrine loop that stimulates CCR3-expressing prostate cells to spread locally and potentially metastasise [6]. Moreover, CCL7 contributes to shaping the immune components of the tumour microenvironment, which can further support tumour growth and progression [59].

Conversely, adiponectin (reduced levels in TBT-PPAT-CM) is often described as a protective factor that counteracts PCa development by directly inhibiting prostate cell proliferation [60,61].

Indeed, TBT exposure was able to either enhance the PPAT-induced increase in prostate cell viability (only in PCa cells, 22Rv1: P1 and P2; DU145: P1, P2, P5; PC3: P3) or stimulate PPAT to acquire this capability (PNT1A: P1 and P3; 22Rv1: P6; DU145: P6; PC3: P4 and P5). TBT (100 nM) was previously shown to directly enhance the proliferation of LNCaP-derived LA16 PCa cells to the same extent as dihydrotestosterone (DHT) [24]. However, to the best of our knowledge, this is the first study to show the potential of TBT-dysregulated human PPAT to increase prostate cell viability, a property previously shown only by us in rat PPAT samples [19].

Although TBT did not affect the potential of PPAT-CM to decrease PCa cell sensitivity to chemotherapeutic drugs in the majority of patients in the study, P2 showed significant results for docetaxel in both DU145 and PC3 and for cabazitaxel in DU145. This observation, once more, raises interest in further exploring the effect of TBT on PPAT-induced resistance to chemotherapy using a larger patient dataset. Another curious observation is that P2 was the least affected by the naïve PPAT secretome in terms of chemotherapeutic drug resistance. This evidence suggests that TBT may not only have the capability to exacerbate pre-existing tumorigenicity but also possesses a pro-tumorigenic potential.

## 5. Conclusions

In conclusion, this study provides preliminary evidence that the secretome of obesogen-dysregulated PPAT can significantly enhance the viability of prostate cells and may have a role in PCa resistance to taxane-based chemotherapy (specifically docetaxel and cabazitaxel). Although the conclusions of the present study must be tempered by some limitations, namely the sample size, the obtained findings demonstrate that human PPAT is a target of obesogenic compounds, highlighting the impact that environmental influences may have on PCa progression. This hypothesis-generating study lays the foundational basis for future work involving larger patient cohorts and aiming at disclosing the molecular pathways underpinning prostate carcinogenesis and resistance to chemotherapy induced by obesogen-dysregulated PPAT. This forthcoming research strategy will allow the identification of new molecular targets for the development of innovative therapeutic approaches in PCa, particularly in obese patients.

## Figures and Tables

**Table 1:** Patients’ information.

Patient No.	Histopathological Diagnosis	Disease Stage	Age	BMI (kg/m^2^)	Classification	Diabetes Type	Anti- Cholesterol or -Diabetic Medication
1	BPH	N.A.	69	28.7	Overweight	N.A.	Rosuvastatin
2	BPH	N.A.	79	31.0	Obese	N.A.	Simvastatin
3	BPH	N.A.	65	30.7	Obese	N.A.	N.A.
4	Carcinoma (GS 6, 3 + 3, ISUP 1)	I	74	24.5	Normal weight	N.A.	Atorvastatin
5	Carcinoma (GS 7, 3 + 4, ISUP 2)	I	60	24.2	Normal weight	N.A.	Atorvastatin Ezetimibe
6	Carcinoma (GS 6, 3 + 3, ISUP 1)	I	82	25.3	Overweight	2	Metformin Dapagliflozin
7	Carcinoma (GS 7, 4 + 3, ISUP 3)	I	68	28.4	Overweight	2	Metformin

BMI: body mass index, BPH: benign prostatic hyperplasia, GS: Gleason score, ISUP: International Society of Urological Pathology grade, N.A.: not applicable.

**Table 2:** Biochemical parameters in patients’ serum.

Patient No.	Total Cholesterol (mg/dL, RV < 200)	LDL (mg/dL, RV < 160)	Triglycerides (mg/dL, RV < 160)	Glucose (mg/dL, RV 70–110)	Total PSA (ng/mL, RV < 4)	Free/ Total PSA (%)
1	144	78	97	105	3.30	14.8
2	177	83	141	98	4.45 (*)	N.P.
3	201 (*)	112	190 (*)	97	4.63 (*)	N.P.
4	134	82	140	90	11.06 (*)	29.8
5	135	57	132	94	8.37 (*)	19
6	154	107	84	102	6.34 (*)	18
7	157	101	105	137 (*)	3.59	N.P.

LDL: low-density lipoprotein, N.P.: not performed, PSA: prostate-specific antigen, RV: reference values. (*) out of reference levels [21,22].

**Table 3:** Maximal increase in the viability of human prostate cells treated with naïve periprostatic adipose tissue secretome.

Patient No.	1	2	3	4	5	6	7
	↑ Cell viability (% relative to DMEM control)						
PNT1A	NS	NS	NS	13	10	17	13
22Rv1	16	10	14	NS	16	NS	14
DU145	13	11	12	12	13	NS	NS
PC3	NS	6	11	NS	NS	15	13

NS: non-significant, ↑: increase.

**Table 4:** Maximal increase in the viability of human prostate cells exposed to tributyltin-treated periprostatic adipose tissue secretome.

Patient No.	1	2	3	4	5	6	7
	↑ Cell viability (% relative to PPAT-CM)						
PNT1A	10	NS	17	NS	NS	NS	NS
22Rv1	15	19	NS	NS	NS	17	NS
DU145	15	23	NS	NS	9	16	NS
PC3	NS	NS	15	8	11	NS	NS

NS: non-significant; PPAT-CM: periprostatic adipose tissue-conditioned media, ↑: increase.

## Data Availability

The original contributions presented in this study are included in the article. Further inquiries can be directed to the corresponding author.
